# Haplotype-resolved chromosome-level genome assembly of Huyou (*Citrus changshanensis*)

**DOI:** 10.1038/s41597-024-03437-3

**Published:** 2024-06-07

**Authors:** Changjiu Miao, Yijing Wu, Lixia Wang, Siqing Zhao, Donald Grierson, Changjie Xu, Wenbo Chen, Kunsong Chen

**Affiliations:** 1 College of Agriculture & Biotechnology, Zhejiang University, Zijingang Campus, Hangzhou, 310058 China; 2 Changshan Agricultural Characteristic Industry Development Center, Quzhou, 324000 China; 3 The State Agriculture Ministry Laboratory of Horticultural Plant Growth and Development, Zhejiang University, Zijingang Campus, Hangzhou, 310058 China; 4 Division of Plant and Crop Sciences, School of Biosciences, University of Nottingham, Sutton Bonington Campus, Loughborough, LE125RD UK; 5 Zhejiang Provincial Key Laboratory of Horticultural Crop Quality Improvement, Zhejiang University, Zijingang Campus, Hangzhou, 310058 China

**Keywords:** Plant genetics, Genome

## Abstract

Huyou (*Citrus changshanensis*) is a significant citrus species that originated in Zhejiang Province, China, where it is also primarily cultivated. It is valued for its distinctive flavor and notable health benefits, owing to its high content of bioactive compounds like naringin and limonin. However, the absence of a high quality reference genome has limited the exploration of these health-promoting compounds in Huyou and hindered research into the mechanisms behind its medicinal properties. In this study, we present a phased chromosome-level genome assembly of Huyou. By combining PacBio and Hi-C sequencing, we generated a primary genome assembly and two haplotypes, comprising nine pseudo-chromosomes, with sizes of 339.91 Mb, 323.51 Mb, and 311.89 Mb, respectively. By integrating transcriptome data and annotations of homologous species, we identified a total of 29,775 protein-coding genes in the genome of Huyou. Additionally, we detected lots of structural variants between the two haplotypes. This represents the first reference genome of Huyou, providing a valuable resource for future studies on its agricultural characteristics and medicinal applications.

## Background & Summary

**Fig. 1:**
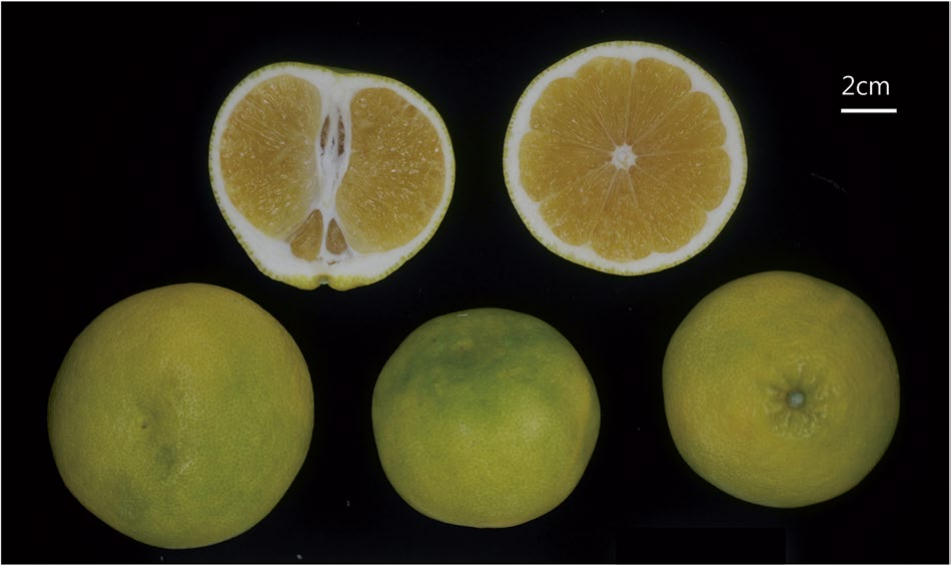
Various views of a Huyou (*Citrus changshanensis*) showing longitudinal section, cross-section, front view, side view, and back view.

Huyou (*Citrus changshanensis* K. S. Chen et C. X. Fu) is a citrus landrace that originated in Changshan County, Zhejiang Province, China, where it has been cultivated for over a century (Fig. 1). Huyou is a natural hybrid with unidentified parents^1,2^. It has a golden skin and a distinctive flavor profile that combines sweet, sour, and a hint of bitterness. This unique taste, along with its high yield and excellent storage capability, allows for extended market availability. Research indicates that bioactive compounds in Huyou, such as naringin and limonin, may offer various health benefits, including hypoglycemic, hepatoprotective, and anti-inflammatory effects^3,4^. These qualities have helped establish Huyou as a key industry in Changshan County. Currently, the Huyou cultivation area in Changshan spans 7,067 hectares, with a total production value exceeding 2 billion Chinese Yuan RMB, providing employment for more than 100,000 people^5^.

Complete genome sequences offer a robust foundation for further molecular and genetic studies. Given their global economic significance, many citrus plants have had their genomes sequenced and published. The first citrus genome to be published was *Citrus sinensis* cv. Valencia^6^, one of the most important sweet oranges, primarily grown for orange juice production. Since then, an increasing number of citrus genomes have been released, including those of *C. grandis*^7^, *C. australis*^8^, and *C. reticulata*^9^, among others. These resources are instrumental in advancing our understanding of citrus evolutionary relationships and agronomic traits.

Citrus is an ancient genus that originated in the south-eastern foothills of the Himalayas around 8 million years ago. Due to extensive interspecific hybridization and long-term domestication, the genus has a complex genetic background and encompasses many members^10^. Some citrus genomes are highly heterogeneous, presenting a significant challenge to genome assembly^9^. Researchers previously used di-haploid lines to reduce assembly complexity, as in the first draft genome of sweet orange (*C. sinensis*)^6^ and the genome of Clementine mandarin (*C. clementina*)^11^. However, with the rapid advancement in sequencing technology and bioinformatics algorithms, these assembly challenges have been gradually overcome, resulting in improved assembly accuracy. Long-read sequencing technologies, such as PacBio and Oxford Nanopore, are now utilized to generate phased genomes^12^, facilitating detailed studies of somatic mutations and allelic gene expression.

Here, we employed an integrated sequencing strategy that combined Illumina short-read sequencing, PacBio long-read sequencing, and Hi-C (high-throughput chromosome conformation capture) sequencing technologies to assemble, annotate, and anchor the Huyou genome at the chromosome level. We used the latest haplotype-resolved software, Hifiasm, to process the long reads and generate high-quality contigs. To construct a chromosome-level genome, we used Juicer and 3D-DNA, and polished the assembly with Illumina short reads. This genome assembly will not only facilitate the identification of important genes related to its agronomic traits but also serve as a valuable resource for medicinal applications.

## Method

### Sample collection

All samples for sequencing were obtained from the Experimental Demonstration Base of Zhejiang University (Changshan) Modern Agricultural Research & Development Center. After sampling, plant materials were ground with liquid nitrogen and stored in a −80 °C freezer for subsequent DNA and RNA extraction and sequencing. Tender leaves from the base of Huyou trees were used for Illumina, HiFi, and Hi-C sequencing in March 2022. To capture comprehensive transcriptome information, we pooled various Huyou tissues, including roots, stems, leaves, flowers, seeds, and mature and immature fruits. Mature fruits were harvested in mid-November 2022. For mature Huyou fruits, we mixed flavedo, albedo, and juice sacs in equal proportions for sequencing. Immature fruits were collected at regular intervals—in mid-June, mid-July, mid-August, and mid-September—and these samples were combined in equal weights.

### DNA and RNA extraction and sequencing

Genomic DNA was isolated from leaf tissue of Huyou using the cetyltrimethylammonium bromide (CTAB) method. The high-quality DNA was then fragmented by g-TUBE (Covaris) and used to create the SMRTbell library using the SMRTbell Template Prep Kit provided by PacBio. Two sequencing cells were performed on PacBio Sequel II system, generating a total of 466.44 Gb of subreads, with ~76 X coverage. After calling consensus sequences from subreads, a total of 25.75 Gb of HiFi (high-fidelity) reads were generated. For Illumina sequencing, libraries were prepared with the NEB DNA Library Rapid Prep Kit, and paired-end sequencing with a read length of 150 bp was performed on the Illumina NovaSeq 6000 platform. After removing adapters and low-quality reads, 22.26 Gb of clean paired-end reads were obtained. The Hi-C libraries were prepared using the Mate-pair Kit and then pair-end sequenced on the Illumina NovaSeq 6000 platform. Following quality control and filtering out low-quality sequences, 216.94 Gb of clean Hi-C reads were generated for downstream analysis (Table 1).

**Table 1:** Statistic of sequencing libraries for Huyou genome assembly.

Library type	Reads number	Clean reads base(bp)	Q30(%)	Estimated coverage
Illumina paired-end	74,731,767	22,256,206,064	93.98	73
PacBio HiFi	1,674,621	25,754,601,493	96.52	84
Hi-C	216,944,309	64,546,299,028	94.09	212

The coverage was calculated with primary assembled genome size of 354.6 Mb.

For RNA extraction, the leaves of the Huyou were processed using the Tengen DP411 kit, while other samples (including roots, stems, flowers, and fruits) were processed using the CLB+ Adderall RN40 kit according to the instruction manual. The mRNA library was constructed using the Dual-mode mRNA Library Prep Kit for Illumina (Hieff NGS Ultima) and subsequently sequenced on the Illumina NovaSeq 6000 platform, generating reads of 150 bp. After filtering, a total of 17.03 Gb reads was remained.

### Genome size and heterozygosity estimation

**Fig. 2:**
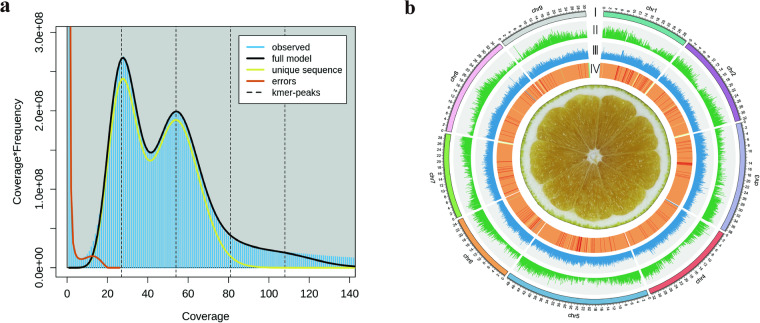
Genome survey and genomic features of Huyou. **(a)** Overview of the 23-mer frequency distribution in the Huyou genome. The X-axis is the k-mer depth, and the Y-axis represents the k-mer frequency for a given depth. **(b)** Genome characteristics of *Citrus changshanensis*. From the outer to the inner layers: Chromosome ideograms for Huyou (Mb scale) (I), Gene density in100-kb windows (II), Repeats density in 100-kb windows (III), GC content (%) (IV).

We calculated the genome size using the formula described in Chen *et al*.^13^, i.e., “Estimated genome size (bp) = total number of k-mer/peak value of k-mer depth distribution”. The analysis started with quality control of the Illumina sequencing reads. We used FastQC v0.11.5^14^ to check the read quality and Trimmomatic v0.38^15^ to trim adapters and remove low-quality sequences. For k-mer frequency-depth distribution, we utilized Jellyfish v2.3.0^16^ to extract and count the k-mers with the parameter “-k 23”. Based on the formula, the estimated genome size of Huyou was 354.6 Mb. Next, we employed GenomeScope v2.0^17^ to estimate the heterozygosity from the k-mer distribution (Fig. 2a). The estimated heterozygosity of Huyou was 3.02%. To compare the heterozygosity of Huyou with other citrus species, we downloaded the whole genome sequence (WGS) data of *C. grandis* and *C. reticulata* from the NCBI (SRR17138707, SRR18687601) and analyzed them with the same method. The estimated heterozygosity rates for *C. grandis* and *C. reticulata* were 1.98% and 1.50%, respectively. These results suggest that Huyou has a relatively high level of heterozygosity compared to other citrus species.

### *De novo* genome assembly

We utilized PacBio long reads and Hi-C reads for genome assembly. Given the high level of heterozygosity in the Huyou genome, we used Hifiasm v0.19.6^18^ in Hi-C mode for assembly and haplotype phasing, which resulted in three sets of contig files: one primary contig file and two haplotype contig files. To eliminate redundant and contamination sequences, we used the following steps. First, we employed Purge_dups v0.0.3 (https://github.com/dfguan/purge_dups) to remove redundant sequences. We then aligned the contigs against the NCBI NT database using BLAST v2.13.0^19^ and removed sequences that had over 80% coverage with non-plant sequences. Additionally, we conducted self-alignment of the contigs using BLAST. Contigs with over 80% similarity and 80% coverage to other contigs were considered as redundant and removed from the contigs files. Finally, we corrected potential sequence errors using uniquely mapped Illumina reads by Pilon v1.23^20^. For the polish of haplotype assemblies, we pooled the two haplotypes together, and aligned Illumina reads to the mixed assembly. The advantage of this strategy was that the reads from the same loci could be accurately assigned to the exact haplotype since the heterozygosity of Huyou was 3.02%. Both the primary assembly and the pooled haplotype assemblies were corrected for three rounds, correcting 115,383 and 144,940 variants, respectively. The primary assembly contained 30 contigs with a total size of 339.91 Mb (Fig. 2b). Haplotype 1 contained 25 contigs with a total size of 323.51 Mb and Haplotype 2 has 30 contigs with a total size of 311.89 Mb. The N50 lengths for the primary assembly, Haplotype 1, and Haplotype 2 were 32.37 Mb, 32.31 Mb and 30.13 Mb, respectively (Table 2).

**Table 2:** Statistic of the genome assembly of Huyou.

Assembly Features	Hap1	Hap2	Primary
Total assembly size (Mb)	323.51	311.89	339.91
Number of contigs	25	30	30
Largest contig (Mb)	49.29	50.29	49.49
Contig N50 (Mb)	32.31	30.13	32.37
GC content (%)	36.11	34.22	36.67
Sequences anchored to chromosomes (Mb)	298.94	297.37	303.56
Complete BUSCO (%)	98.0	98.2	98.0
LAI index	15.40	19.54	18.63
QV	42.74	43.36	43.26
k-mer completeness	96.87%		

**Fig. 3:**
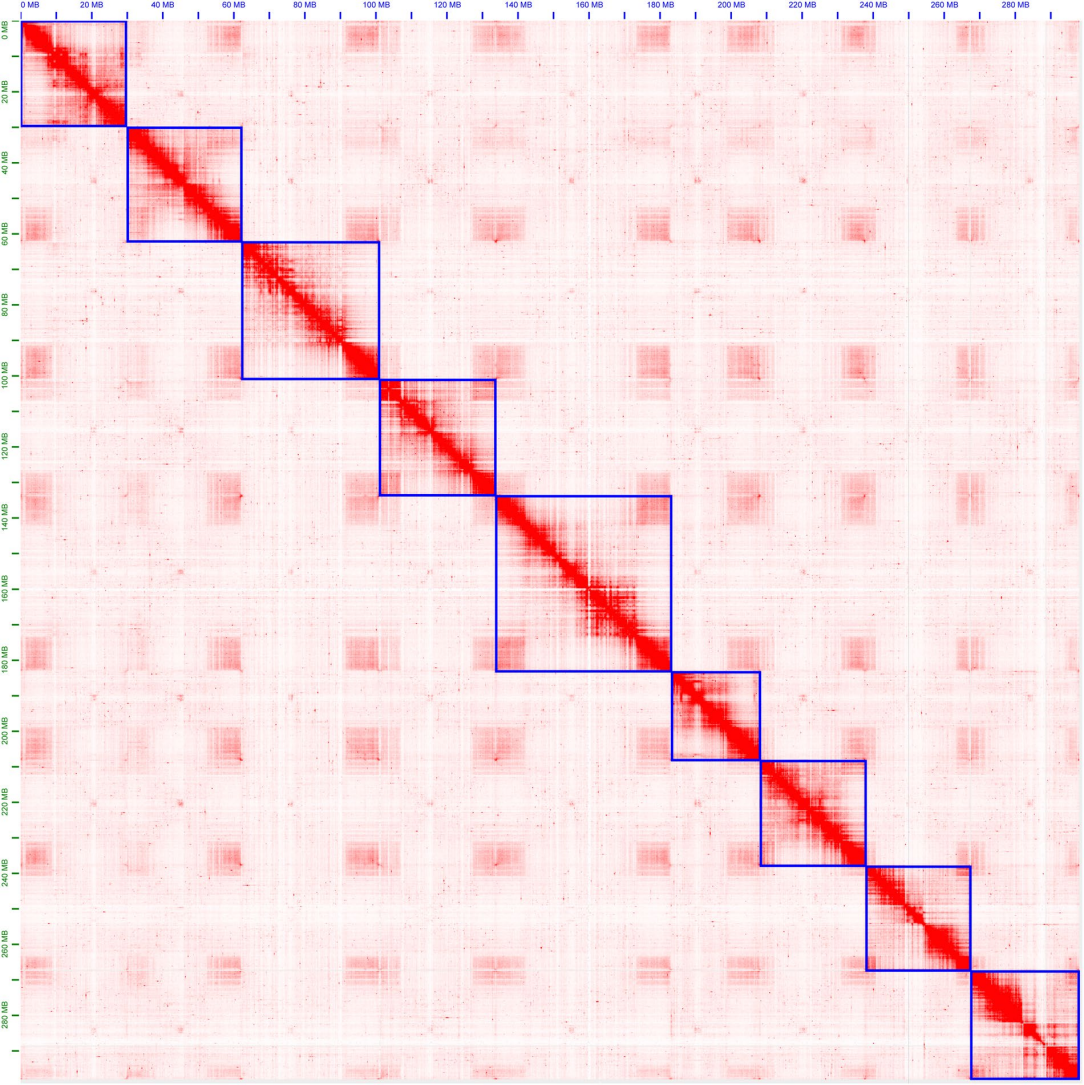
Hi-C interactive heatmap of Huyou primary genome assembly.

We used 80 Gb Hi-C reads to anchor the contigs into the psuedo-chromosomes. Juicer v1.6^21^ was employed to map the Hi-C reads to the draft assembly, and 3D-DNA (version 180114)^22^ was used to generate a chromosome-level assembly. Visualization of the Hi-C contact map was done using Juicebox v1.11.08 (https://github.com/aidenlab/Juicebox, v1.1108), allowing for manual adjustments and corrections (Fig. 3 and Supplementary Figure 1). Finally, we successfully anchored 307.15 Mb sequences into 9 psuedo-chromosomes for the primary genome assembly, accounting for 89% of the total genome size. The N50 of the chromosome-level assembly was 32.27 Mb. Using the same pipeline, we anchored 298.94 Mb and 297.36 Mb of sequences to the pseudo-chromosomes for the two haplotypes, respectively.

Since Huyou is a natural hybrid between two citrus species, we used phylogenetic trees to phase each psuedo-chromosome in each haplotype. We constructed phylogenetic trees for each psuedo-chromosome with other five citrus species, including *C. grandis*, *C. sinensis*, *C. reticulata*, *C. medica*, *C. clementina*, and *Atalantia buxfoliata* as outgroup. OrthorFinder^23^ was used to identify single-copy homologous genes, which were then used to build the trees. Multiple sequence alignments of these single-copy genes were performed using Muscle^24^, followed by filtering with trimAl v1.4^25^ to remove sequences that were absent in more than 20% of these species mentioned above. The resulting files were then utilized to construct phylogenetic trees by IQ-TREE v2.2.2.3^26^ with the parameter “ -m MFP -B 1000 -alrt 1000 ”. Based on the phylogenetic trees, the nine psuedo-chromosomes claded with pummelo (*C. grandis*) were grouped together and named as Hap1 (Supplementary Figures 2–10). The other nine psuedo-chromosomes were grouped together and named as Hap2. These results support the hypothesis that Huyou is a hybrid of two citrus species.

### Repeat annotation

We used the Repeat_Library_Construction-Advance process from the Maker homepage (https://weatherby.genetics.utah.edu/MAKER/wiki/index.php/Repeat_Library_Construction-Advanced) to identify repetitive sequences in the Huyou genome. First, we identified MITE and LTR repeat sequences in the genome using MITE-Hunter v8.28^27^ and LTR-Retriver v2.9.0^28^. We then used RepeatModeler v2.0.1^29^ to construct a *de novo* repetitive library. Finally, we utilized the ProtExluder.pl script from Repeat_Library_Construction-Advance to compare the repetitive library sequences with the plant protein database and removed the sequences that were likely to be protein coding genes. Subsequently, we obtained the repetitive library of Huyou and used RepeatMasker v4.1.2^30^ to annotate the genome assembly. Overall, 52.17% of the Huyou’s primary genome was identified as repetitive sequences (Table 3). The most abundant repeats are LTRs, consistent with other citrus species.

**Table 3:** Percentage of repetitive elements in Huyou genome compared with representative Citrus species.

Repeats type		*Citrus changshanensis* (Primary)	*Citrus changshanensis* (Hap1)	*Citrus changshanensis* (Hap2)	*Citrus australis* v1.0	*Citrus clementina* cv. Clemenules v1.0	*Citrus sinensis* cv. Ridge Pineapple v1.1	*Citrus maxima* cv. Cupi Majiayou v1.0
Retroelements	LTR	18.09	17.76	18.43	21.01	19.96	15.65	30.79
	SINE	0.02	0.02	0.02	0	0.03	0	0.32
	LINE	1.38	1.40	1.45	1.66	1.24	1.41	1.65
DNA transposons	MITE	5.94	5.93	6.23	3.38	2.69	1.68	7.73
	other DNA transposons	3.37	3.36	3.5	0			
Rolling-circles		0.39	0.37	0.42	0.01	0	0.04	0
Unclassified		22.34	21.17	17.82	25.95	18.62	10.26	3.09
Small RNA		0.2	0.2	0.64	0.1	0.02	0	1.91
Simple repeats		0.03	0.03	0.03	0.96	0.5	10.26	1.91
Total repeats		52.17	50.24	48.54	53.17	44.67	31.06	45.83

### Protein-coding gene prediction

Evidence for gene prediction included RNA-Seq data from Huyou and homologous protein sequences. For transcriptome evidence, we aligned the RNA-seq reads to the repeat-masked genome using Hisat2 v2.2.1^31^, and then assembled the transcripts using Scallop v1.1.2^32^ with the parameters “–library_type unstranded–min_transcript_coverage 0.1–min_single_exon_coverage 10–min_num_hits_in_bundle 10–min_transcript_length _base 200–min_bundle_gap 20”.

For protein evidence, we downloaded proteomic sequences of four closely related citrus including *C. sinensis*, *C. reticulata*, and *C. grandis* (cv.‘Cupi Majiayou’ and cv. ‘Wanbaiyou’ v1.0) from the Citrus Pan-genome to Breeding Database (http://citrus.hzau.edu.cn/download.php). We also downloaded SwissProt protein sequences from UniProt protein databases (https://www.uniprot.org) for gene annotation.

For *ab initio* gene prediction, we used BRAKE v2.1.4^33^, which combines results from two gene prediction programs, GENEMARK and AUGUSTUS. We trained the prediction model of BRAKE using the bam file of the RNA-Seq generated in the previous step.

Maker^34^ integrated the *ab initio* prediction and all those evidences to predict the genes in the genome assembly. A total of 29,775 genes were successfully predicted in the primary genome. We applied the same method to predict protein-coding genes in the two haplotypes, resulting 29,716 and 29,806 protein-coding genes, respectively (Table 4).

**Table 4:** Statistics of predicted protein-coding genes in Huyou genome compared to representative Citrus species.

Features	*Citrus changshanensis* (Primary)	*Citrus changshanensis* (Hap1)	*Citrus changshanensis* (Hap2)	*Citrus australis* v1.0	*Citrus clementina* cv. Clemenules v1.0	*Citrus sinensis* cv. Ridge Pineapple v1.1	*Citrus maxima* cv. Cupi Majiayou v1.0
Number of genes	29,775	29,716	29,806	29,464	24,533	25,379	30,123
Mean gene length (bp)	2,785.72	2,757.11	2,705.71	2,846.03	3,044.79	2,766.64	3,253.12
Mean exon number per gene	4.9	4.9	4.9	6.40	5.30	4.90	8.07
Mean exon length (bp)	257.41	256.09	255.05	210.75	288.10	268.87	276.20
Number of transcripts	29,775	29,716	29,806	32,009	33,929	46,147	42,886
Mean transcript length (bp)	1,255.89	1,246.49	1,258.52	1,239.76	1,707.63	1,761.75	1,571.77
Mean CDS length (bp)	1,197.26	1,192.95	1,186.29	1,130.00	1,242.46	1,244.85	1,141.10
Protein Busco (%)	94.2	94.9	95.2	95.4	95.6	87.5	95.4

### Gene function annotation

To annotate gene functions, we used BLAST to compare all the protein sequences of Huyou against the protein databases of *C. grandis*, *C. reticulata*, *C. medica*, *C. ichangensis*, *C. clementina*, *Solanum lycopersicum*, *Arabidopsis thaliana* as well as Swissprot database. We run Blastp v2.2.26 with the parameters “-e 0.0001 -v 200 -b 200 -m 0 -a 8”. The output files were processed with AHRD v3.3.3 (https://github.com/groupschoof/AHRD) to generate readable function descriptions, with weights assigned as follows: 100 for citrus plant proteins, 80 for SwissProt proteins, and 50 each for *S. lycopersicum* and *A. thaliana* proteins. Additionally, we used InterProScan^35^ to annotate the domains and GO terms in these genes. As a result, 90.21% of the genes were assigned domain annotations, and 59.27% of the genes assigned GO annotations (Table 5).

**Table 5:** Statistic of functional annotation.

DataBase	Hit Number	Percentage (%)
*Arabidopsis thaliana*	24,521	82.35
*Solanum lycopersicum*	24,953	83.81
SwissProt	21,825	73.30
Citrus genus	28,845	96.88
Interproscan domain	26,861	90.21
GO term	17,648	59.27

### Structural variations between two haplotypes

**Fig. 4:**
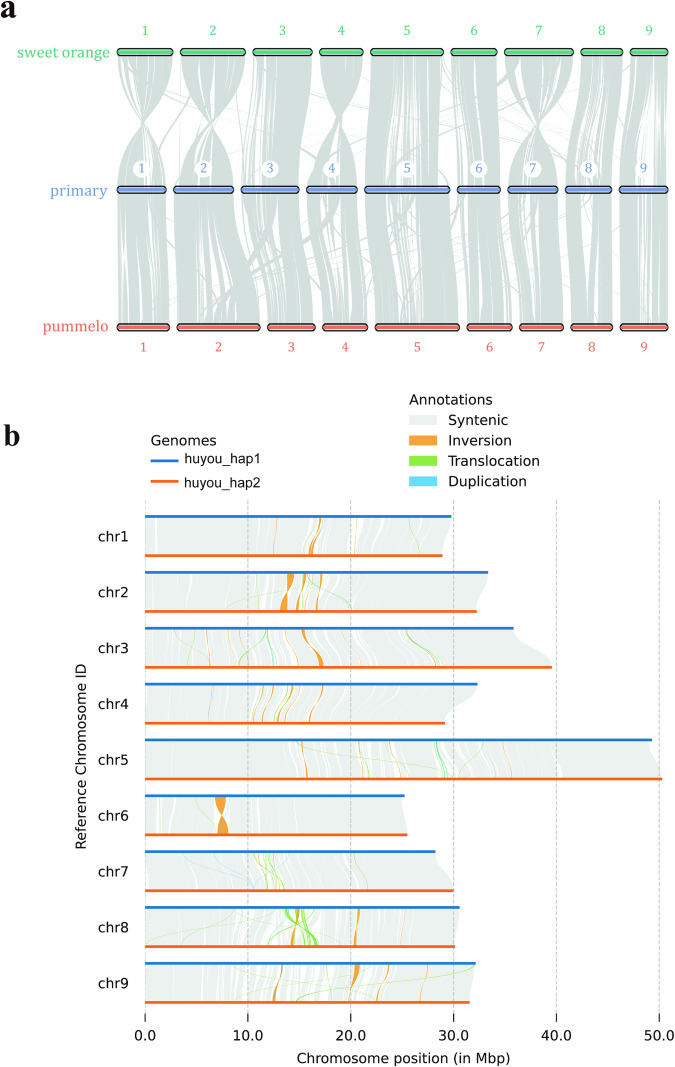
The sequence collinearity. **(a)** The sequence collinearity and structural variants between Hap1 and Hap2. **(b)** The collinearity between Huyou primary genome assembly, sweet orange (*C. sinensis*) and pummelo (*C. grandis*).

We identified structural variations between two haplotypes of Huyou using the following steps. We aligned the genome assemblies of the two haplotypes using minimap2 v2.24^36^, with parameters ‘-ax asm5–eqx’. SyRI v1.6.3^37^ was used for identifying structural variants between two haplotypes with default parameters. Poltsr v1.1.1^38^ was used for generating synteny plot (Fig. 4a). This analysis yielded a total of 1,950,035 single-nucleotide polymorphism (SNP) differences and 253,554 insertion-deletions (InDels), including 122,153 insertions and 131,401 deletions. SyRI detected 454 duplications (DUPs) with a total length of 2.2 Mb, 78 inversions (INVs) with a total length of 4.75 Mb, and 545 translocations (TRANSs) with a total length of 3.23 Mb.

## Data Records

The whole-genome sequencing data (Illumina genomic sequencing reads, PacBio HiFi reads, Hi-C data, and RNA-Seq reads) were deposited to the NCBI Sequence Read Archive with accession number SRP497450^39^. The genome assembly data had been submitted to GenBank with accession number JBCGBQ000000000^40^. The genome annotation GFF, CDS sequences and protein sequences are available under accession number GWHEQVQ00000000.1 that is publicly accessible at https://ngdc.cncb.ac.cn/gwh^41,42^.

## Technical Validation

### High collinearity with other citrus genomes

We analyzed the collinearity of the primary genome assembly of Huyou with both pummelo and sweet orange and obtained the results shown in Fig. 4b. Huyou shares 128 blocks containing 20,327 homologous gene pairs with pummelo, and 185 blocks containing 19,332 homologous gene pairs with sweet orange. The collinearity of two haplotype assemblies of Huyou with pummelo and sweet orange are shown in Supplementary Figure 11.

### High completeness of genome assembly

We used BUSCO v5.3.2^43^ to assess the completeness of the genome assembly by comparing it with the conserved single copy orthologue genes from the eudicots_odb10 database. The analysis revealed that the primary assembly had a completeness of 98.0%, while Hap1 and Hap2 had completeness of 98.2% and 98.0%, respectively. To evaluate the completeness of the genome annotation, we extracted the protein sequences from the gene GFF3 file using GFFread v0.12.1^44^. These protein sequences were then compared with the eudicots_odb10 protein database. The results indicated that the primary assembly annotation achieved a completeness of 94.2%, while Hap1 and Hap2 exhibited annotation completeness of 94.9% and 95.2%, respectively (Table 4). To evaluate genome quality, we calculated LTR Assembly Index (LAI). First, we identified LTRs using LTRharvest^45^ and LTR_FINDER v1.1^46^, then calculated the LAI indices for the three assemblies using LTR_retriever^28^. The LAI indices for the three genomes were 15.40, 19.54 and 18.63, respectively (Table 2). We also used Merqury v1.3^47^ to assess assembly quality by aligning Illumina sequence reads to the genome to calculate Quality Value (QV) and k-mer completeness. The QV and k-mer completeness of primary, Hap1 and Hap2 are shown in Table 2. These evaluations suggest that the genome assemblies are of high quality and suitable for use as reference genomes.

### Supplementary information

Supplementary figures

## Data Availability

If no detailed parameters were mentioned, all software and tools in this study were used with their default parameters. No specific code or script was used in this study.
