# Supplementary material for: Haplotype-resolved chromosome-level genome assembly of Huyou (Citrus changshanensis)
Source: Sci Data. 2024 Jun 7;11:605. doi: 10.1038/s41597-024-03437-3 (PMC11161639; doi:10.1038/s41597-024-03437-3)
Supplement: Supplementary file 1 — Supplementary figures [file 41597_2024_3437_MOESM1_ESM.docx]

[**Supplementary Figure 1** Hi-C interactive heatmaps. 3](#_Toc166502488)

[**Supplementary Figure 2** Phylogenetic tree of psuedo-chromosomes 1. 4](#_Toc166502489)

[**Supplementary Figure 3** Phylogenetic tree of psuedo-chromosomes 2. 4](#_Toc166502490)

[**Supplementary Figure 4** Phylogenetic tree of psuedo-chromosomes 3. 5](#_Toc166502491)

[**Supplementary Figure 5** Phylogenetic tree of psuedo-chromosomes 4. 5](#_Toc166502492)

[**Supplementary Figure 6** Phylogenetic tree of psuedo-chromosomes 5. 6](#_Toc166502493)

[**Supplementary Figure 7** Phylogenetic tree of psuedo-chromosomes 6. 6](#_Toc166502494)

[**Supplementary Figure 8** Phylogenetic tree of psuedo-chromosomes 7. 7](#_Toc166502495)

[**Supplementary Figure 9** Phylogenetic tree of psuedo-chromosomes 8. 7](#_Toc166502496)

[**Supplementary Figure 10** Phylogenetic tree of psuedo-chromosomes 9. 8](#_Toc166502497)

[**Supplementary Figure 11** The genome collinearity. . 9](#_Toc166502498)

**A**


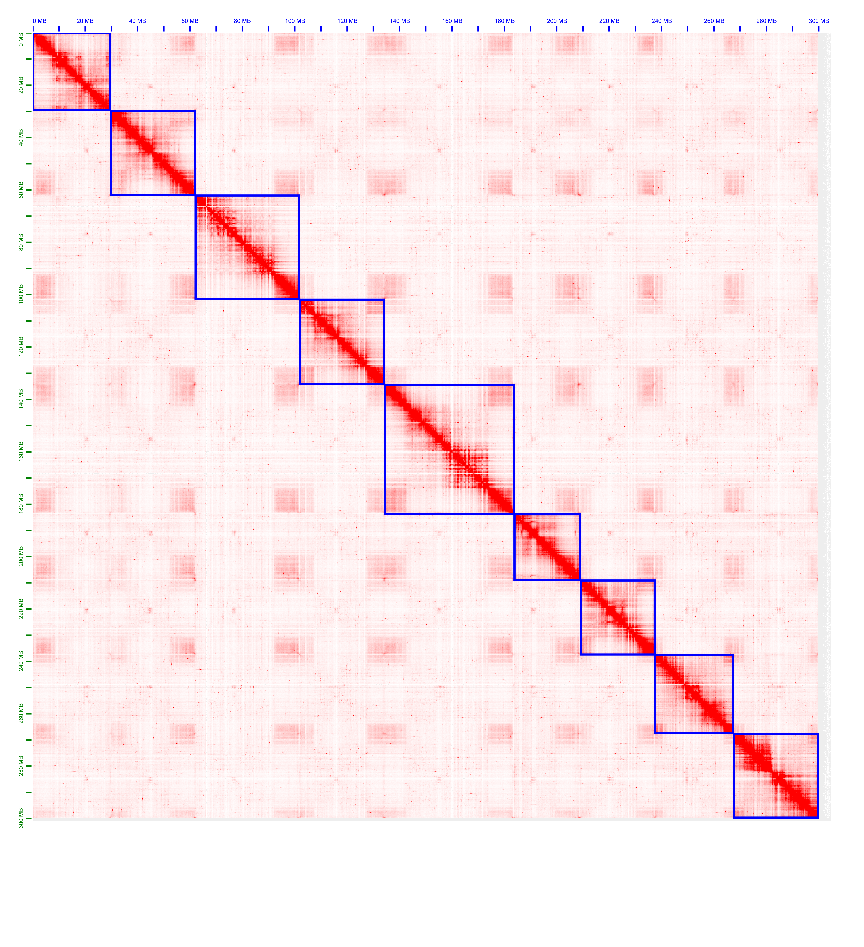


**B**


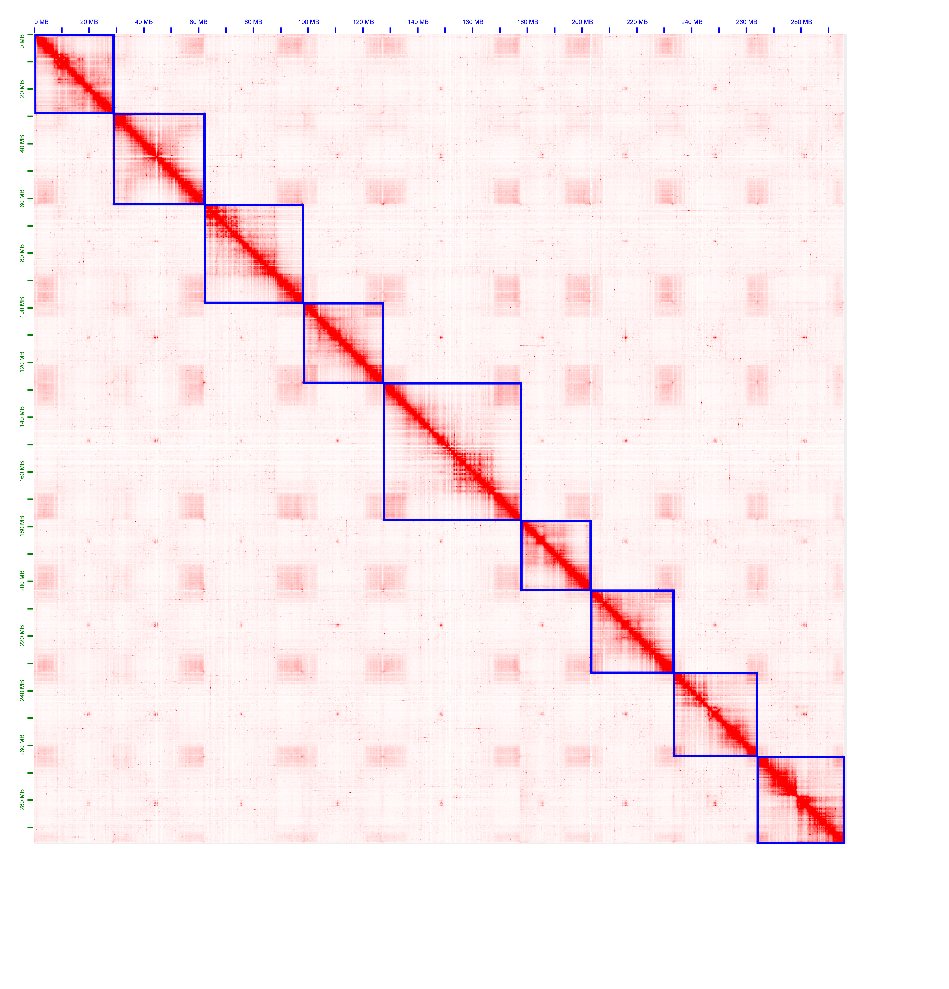


**C**


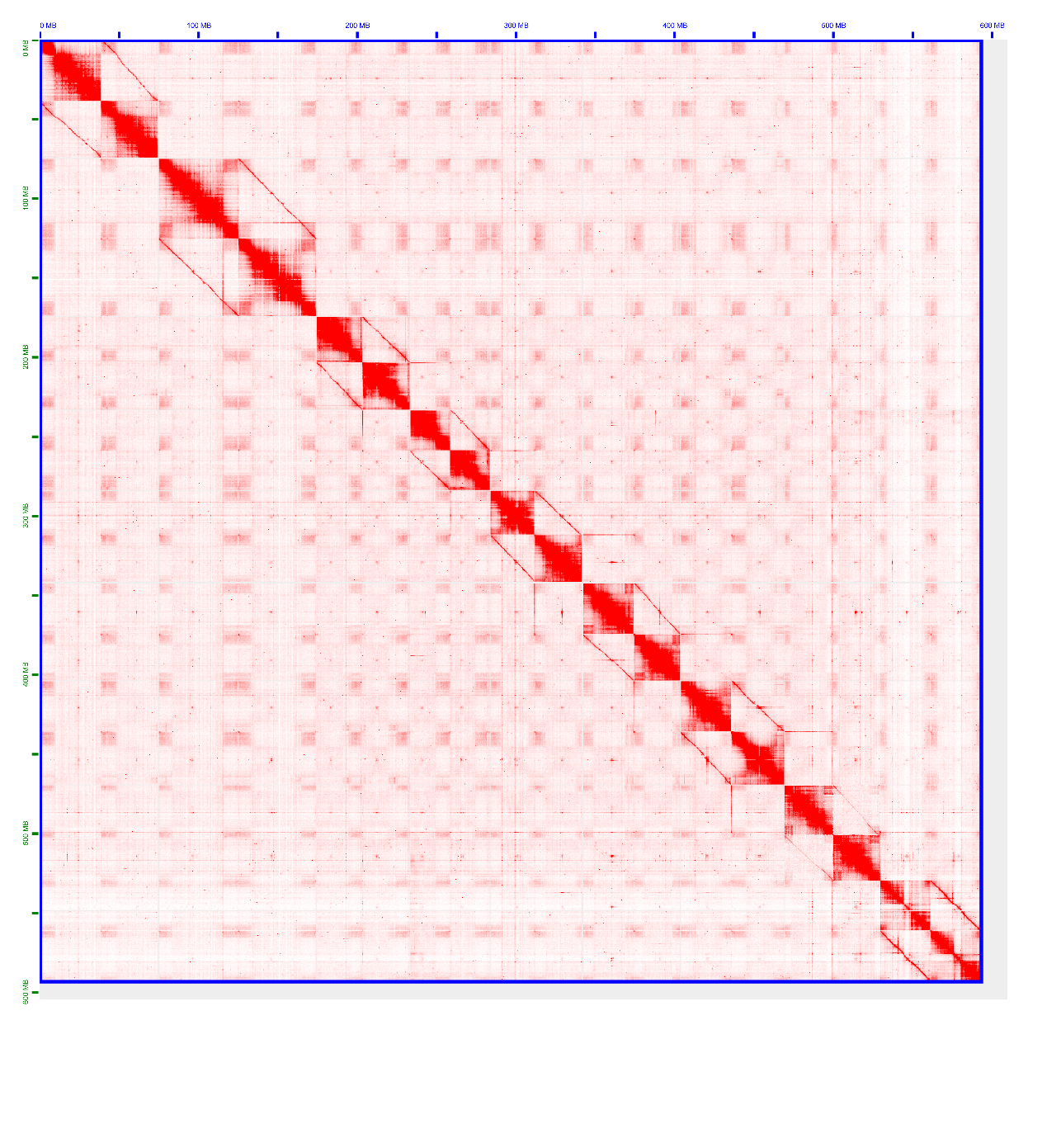


## **Supplementary Figure 1** Hi-C interactive heatmap of Huyou’s haplotype 1(**A**) , haplotype 2 (**B**), and between the two haplogypes **(C)**.


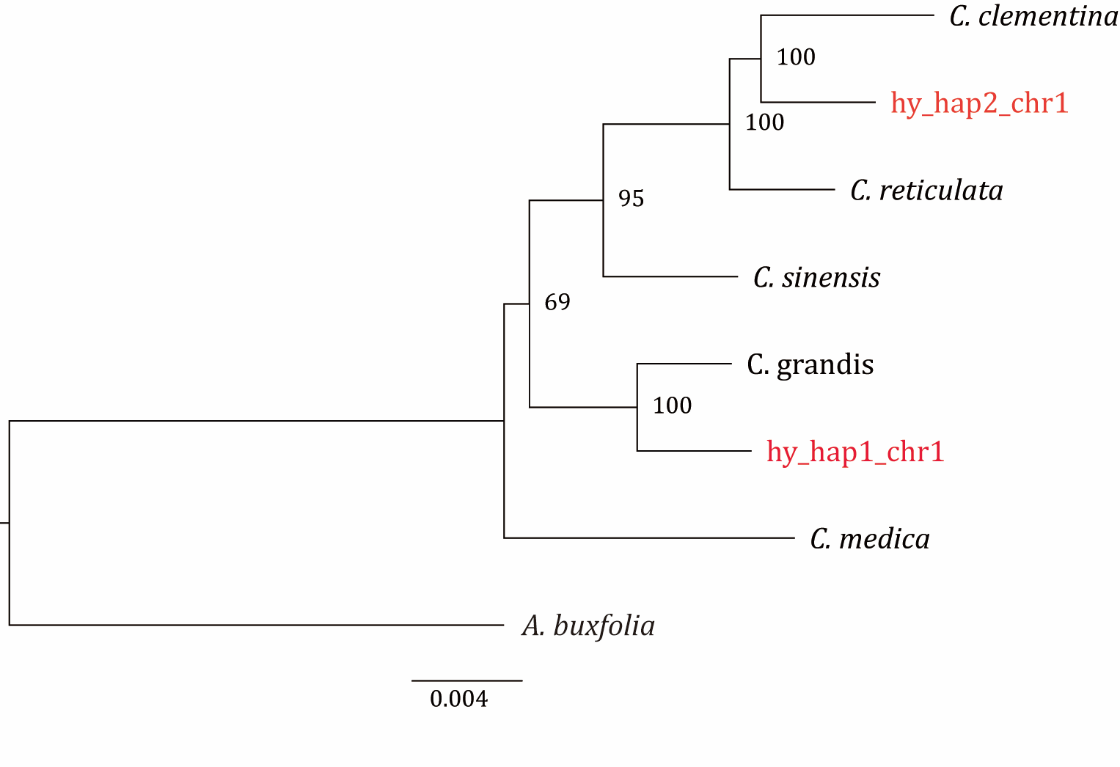


## **Supplementary Figure 2** Phylogenetic tree of psuedo-chromosomes 1.


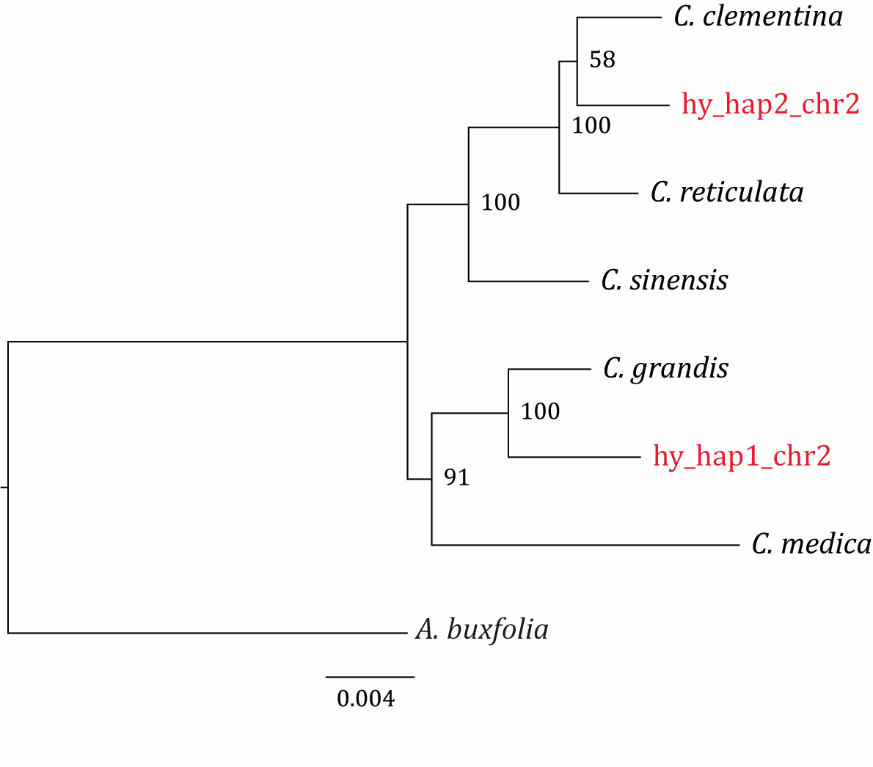


## **Supplementary Figure 3** Phylogenetic tree of psuedo-chromosomes 2.


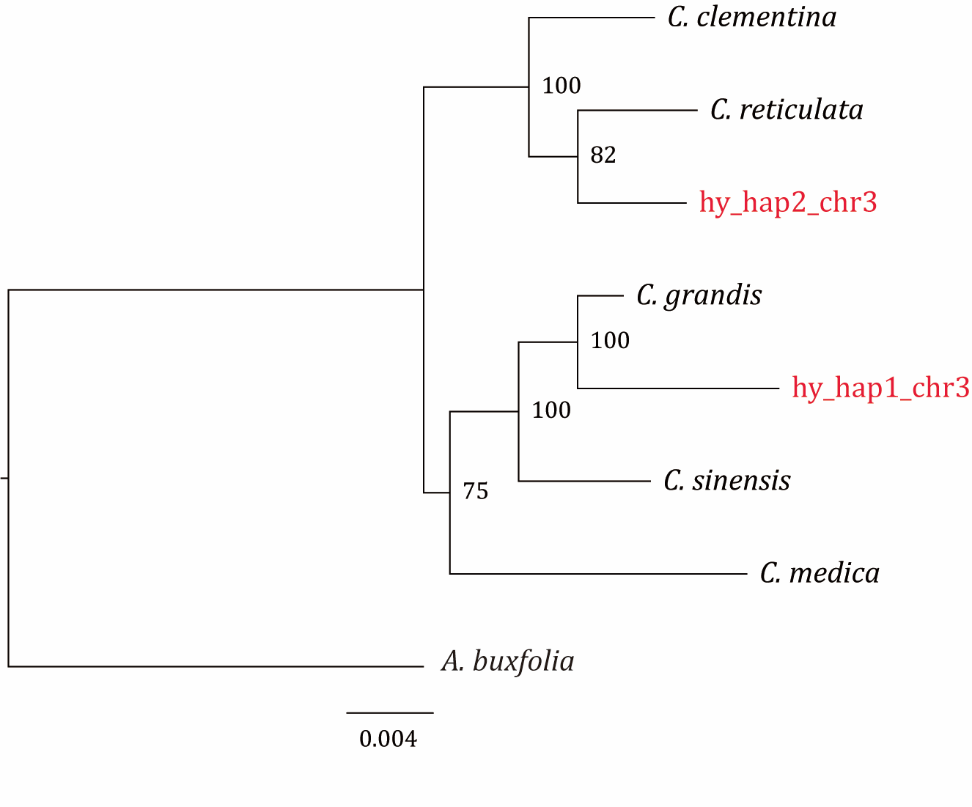


## **Supplementary Figure 4** Phylogenetic tree of psuedo-chromosomes 3.


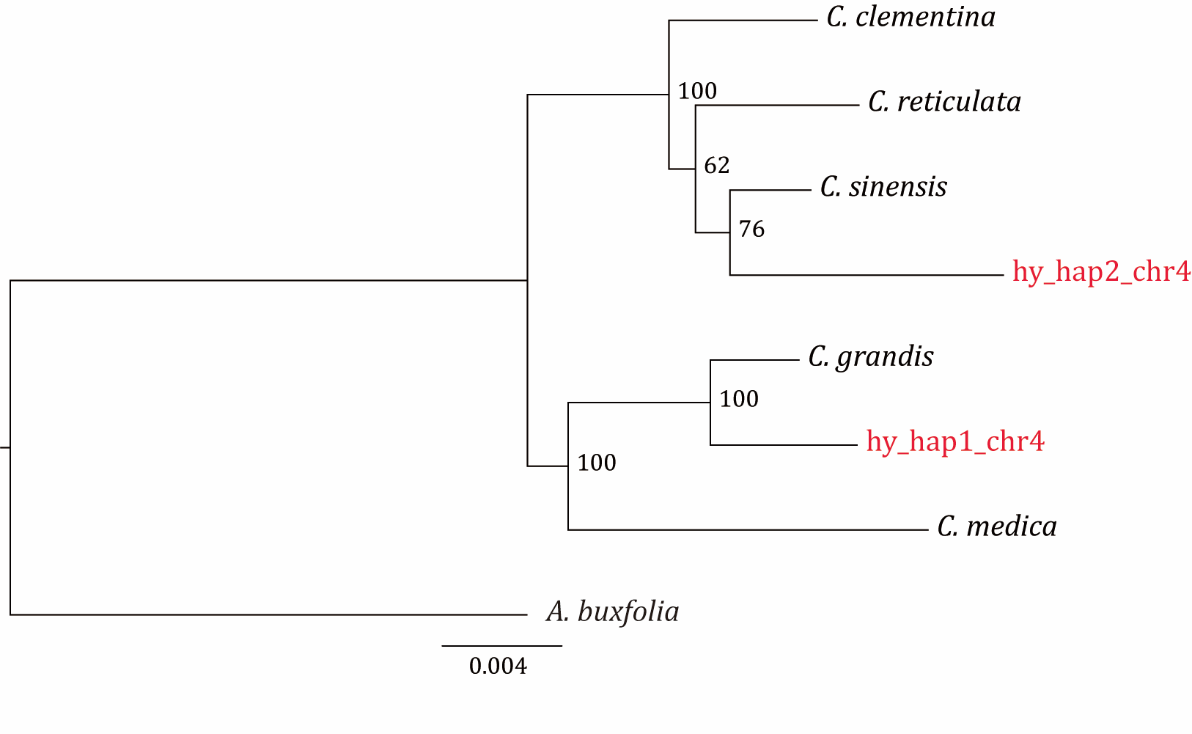


## **Supplementary Figure 5** Phylogenetic tree of psuedo-chromosomes 4.


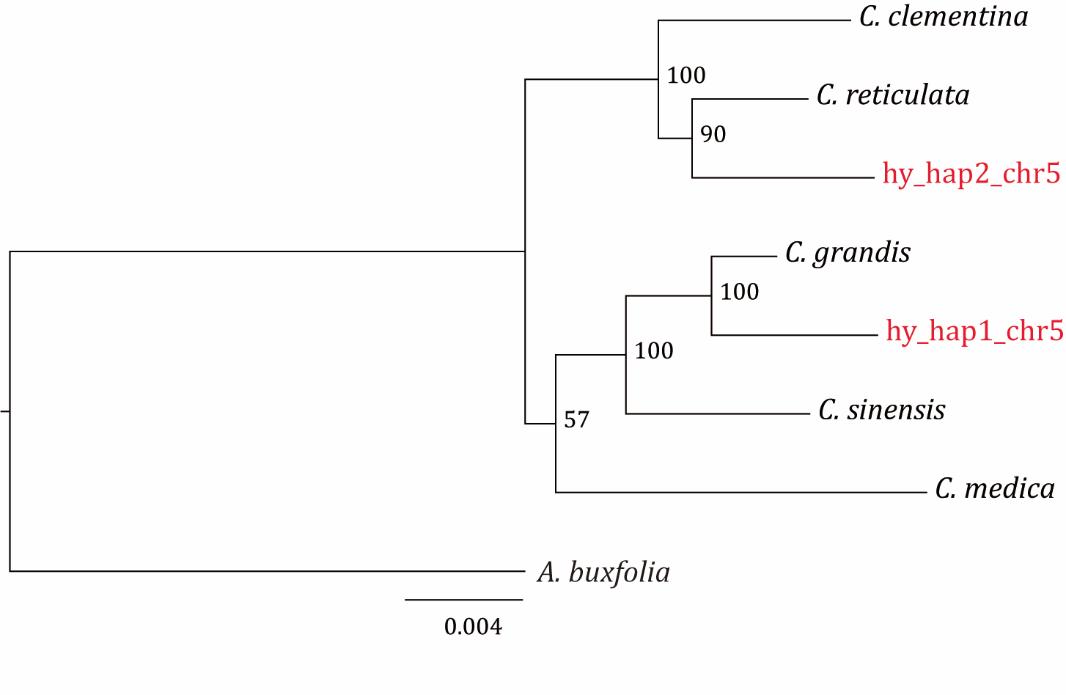


## **Supplementary Figure 6** Phylogenetic tree of psuedo-chromosomes 5.


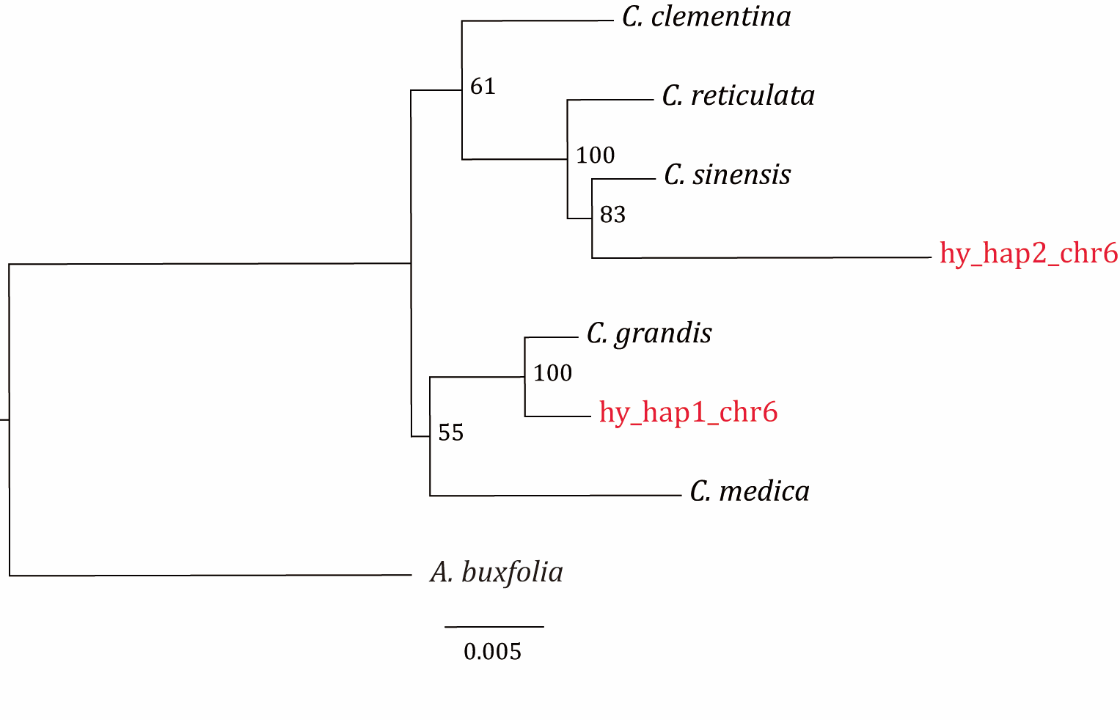


## **Supplementary Figure 7** Phylogenetic tree of psuedo-chromosomes 6.


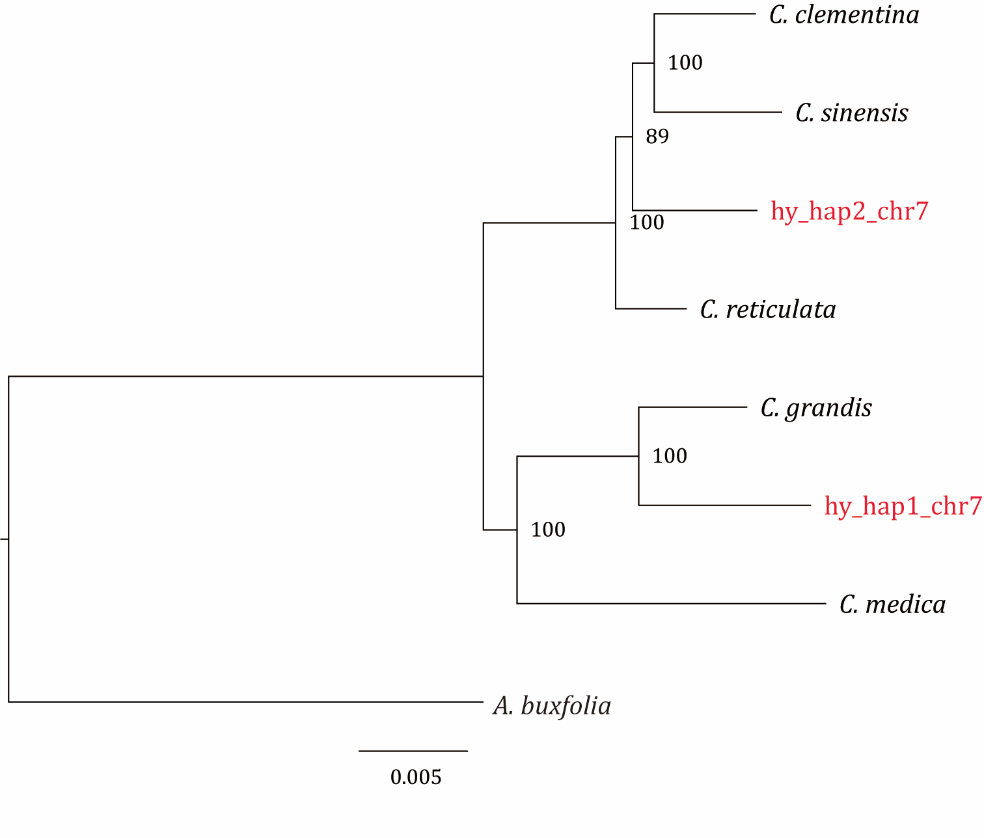


## **Supplementary Figure 8** Phylogenetic tree of psuedo-chromosomes 7.


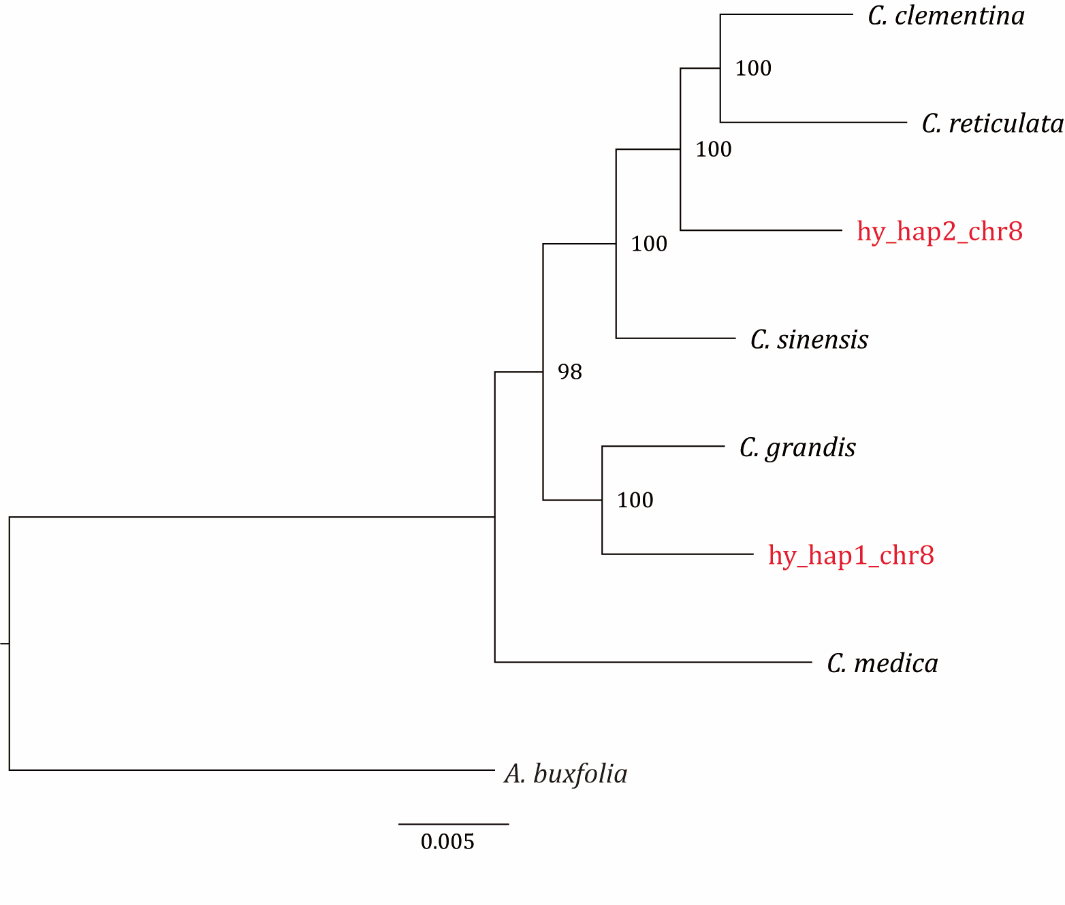


## **Supplementary Figure 9** Phylogenetic tree of psuedo-chromosomes 8.


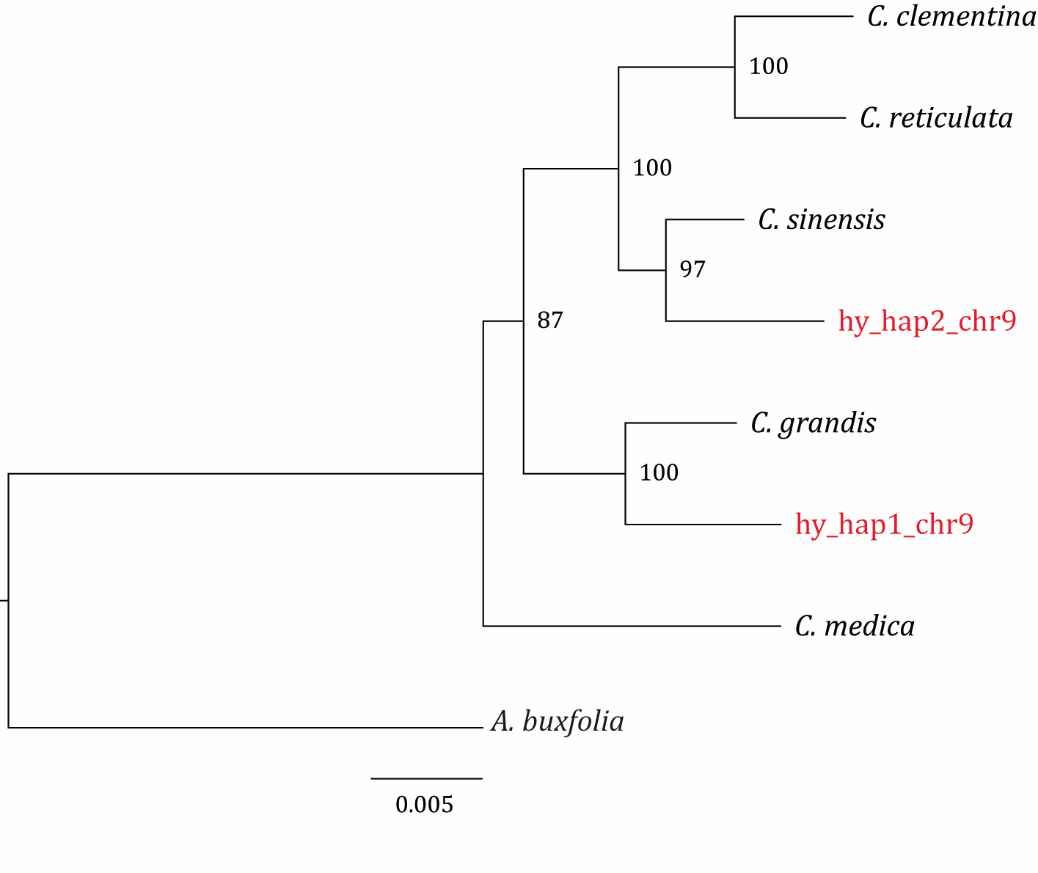


## **Supplementary Figure 10** Phylogenetic tree of psuedo-chromosomes 9.

**A**


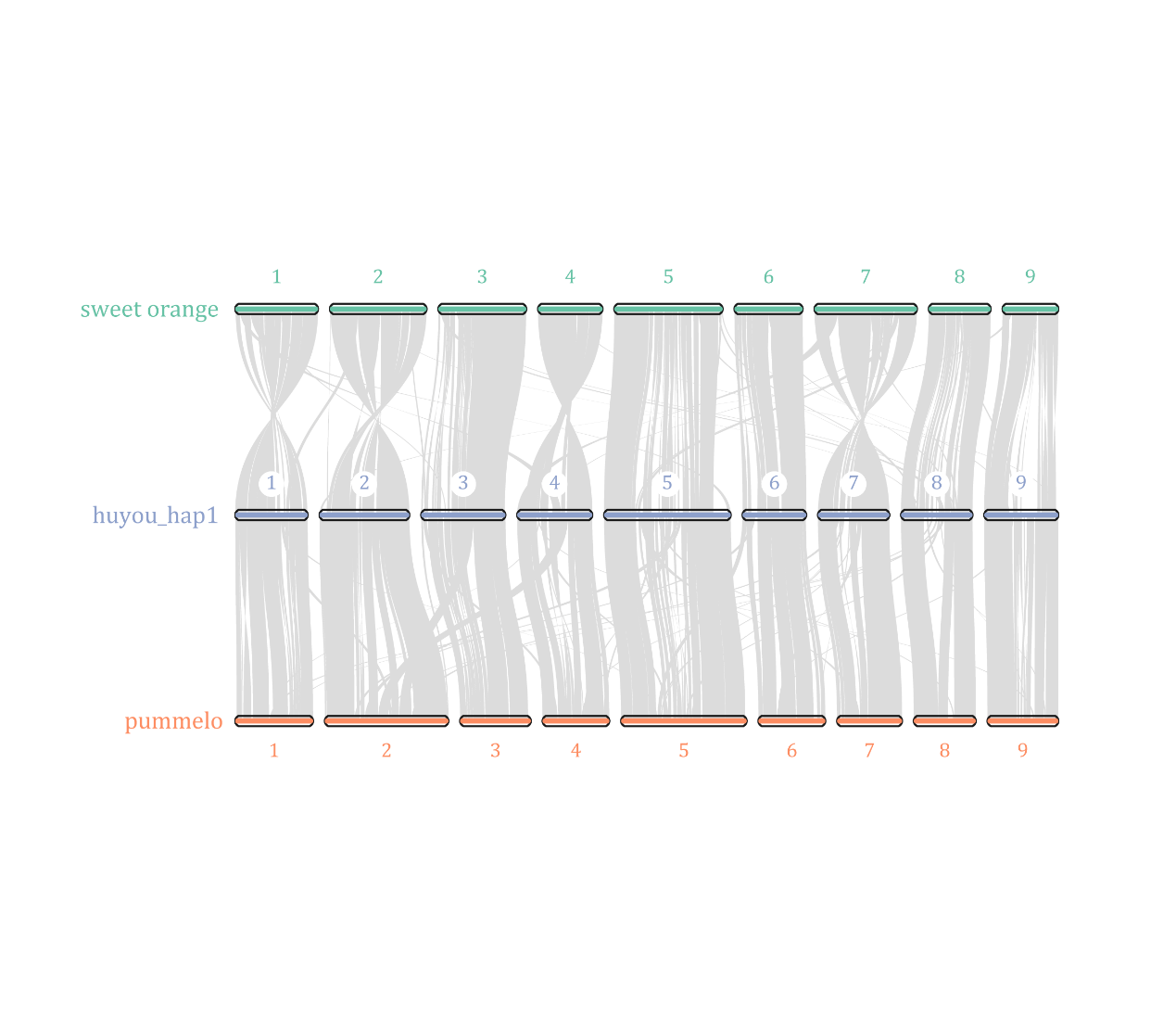


**B**


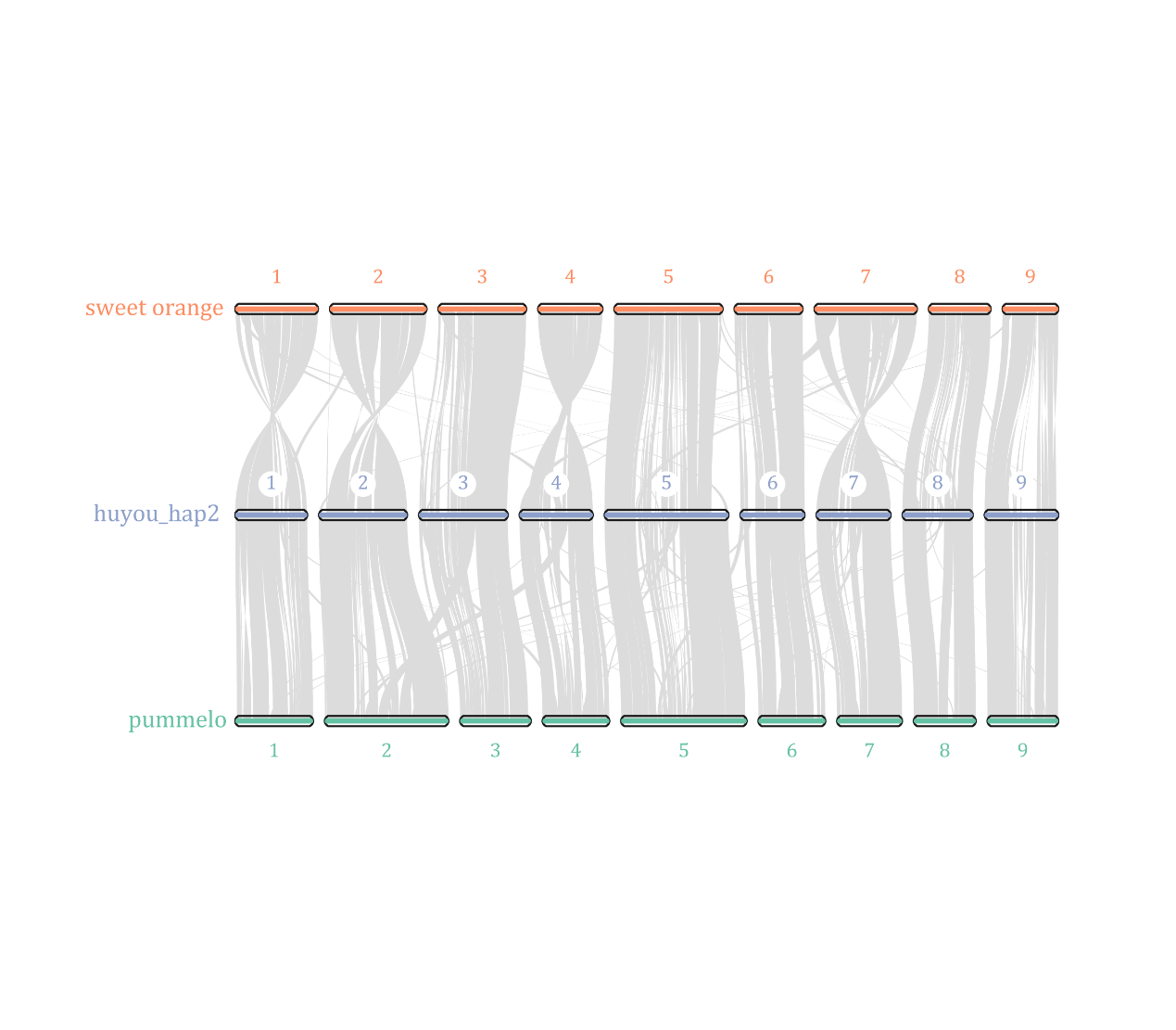


## **Supplementary Figure 11** The genome collinearity. (**A**) Collinearity among Huyou’s haplotype 1, sweet orange (*C. sinensis*) and pummelo (*C. grandis*). (**B**) Collinearity among Huyou’s haplotype 2, sweet orange (*C. sinensis*) and pummelo (*C. grandis*).
